# Supplementary material for: MELODY trial: study protocol for a 12-week randomised controlled trial of adjunctive melatonin, digital cognitive behavioural therapy for insomnia or pill placebo to improve depressive symptoms in young adults with mood disorders
Source: BMJ Open. 2026 Aug 24;16(8):e114817. doi: 10.1136/bmjopen-2025-114817 (PMC13504852; doi:10.1136/bmjopen-2025-114817)
Supplement: Supplementary file 1 [file bmjopen-16-8-s002.pdf]

THE UNIVERSITY OF SYDNEY
Brain and Mind Centre

Consent Form

**Title**	MELODY Trial: A 12-week randomised controlled trial of adjunctive melatonin or digital cognitive behavioural therapy for insomnia, or placebo to improve depressive symptoms in young adults with mood disorders
**Short Title**	The MELODY Trial
**Protocol Number**	X23-0450
**Project Sponsor**	The University of Sydney
**Principal Investigator**	Professor Ian B. Hickie
**Location**	Brain and Mind Centre, University of Sydney<br>The Woolcock Institute of Medical Research

Consent Agreement

I have read the Participant Information Sheet, or someone has read it to me in a language that I understand.

I understand the purposes, procedures and risks of the research described in the project.

I understand that the following is involved in this study:

- Biological samples and other health information will be collected
- I will participate in health surveys and questionnaires
- My research records may be accessed by authorised researchers
- I will not receive any routine results or care from the study team
- Data gathered during the study may be published, provided that I cannot be identified
- I will not receive or be able to claim any payment, compensation, royalty or any other financial benefit from this study, other than as described in Section 5.

I have had an opportunity to ask questions, and I am satisfied with the answers I have received.

I freely agree to participate in this research project as described.

I understand that I am free to withdraw at any time during the study and withdrawal will not affect my relationship with any of the organisations and/or study team members.

I understand that I will be given a copy of this document to keep.

I understand that, if I decide to discontinue the study, I may be asked to attend follow-up visits to allow collection of information. I can refuse to do this. I can also request that my data be completely deleted from the study.

I agree to participate in this research understanding that my anonymised data may be stored and shared for future research purposes, subject to appropriate privacy restrictions.

I give permission for my treating clinicians, other health professionals, hospitals or laboratories to release information to Brain and Mind Centre, University of Sydney, concerning my condition and treatment for the purposes of this study. I understand that such information will remain confidential.

I consent to my information being provided, only when necessary, to the relevant authorised services such as couriers, laboratories, your treating clinicians, Woolcock Institute, and ambulatory services for the operational purposes of the study, if required.

I understand the activities and requirements of the sub-study and am free to accept or decline participation according to my own personal preference.

In addition, I also give consent for the following optional activities:

	Consent of participant
I consent my blood samples to be collected and sent to local pathology centres for analyses at baseline and the end of the treatment.	☐ Yes ☐ No
I express interest to participate in the sub-study (overnight sleep/circadian assessments) and understand that I will be asked to provide consent separately for the sub-study	☐ Yes ☐ No
I give consent for my health information to be used for future research purposes	☐ Yes ☐ No
I would like to receive an overall summary of the results of this current study (via newsletter) once they are made available:	☐ Yes ☐ No<br>If yes, please provide email address:<br>______________________________

By signing below, I consent to the use of my saliva samples for genomic research as described in Section 10 of the Participant Information Sheet. I understand that my DNA may be studied as part of this study.

I understand that my anonymous clinical information and the biological samples (including DNA) I have provided may be made available to authorised researchers in the future, some of whom may have commercial interests. I donate my clinical information and biological samples freely for these purposes and waive any claim to commercial rights arising from this work.

In respect to the storage and use of my genetic samples, I give permission for the use of my DNA for the purpose of:

1. This specific research study ☐ Yes ☐ No
2. Closely-related research projects (specified research) ☐ Yes ☐ No
3. Any future research projects (unspecified research) ☐ Yes ☐ No

Declaration by Participant – for participants who have read the information

Name of Participant (please print) ______________________________

Signature ____________________ Date ____________________

Declaration by Study Doctor/Senior Researcher†

I have given a verbal explanation of the research project; its procedures and risks and I believe that the participant has understood that explanation.

Name of Study Doctor/
Senior Researcher† (please print) ______________________________

Signature ____________________ Date ____________________

† A senior member of the study team must provide the explanation of, and information concerning, the research project.

Note: All parties signing the consent section must date their own signature.

The follow section is to be signed by participants who are invited and agreed to take part in sub-study, in addition to the consent above.

Declaration by Participants for Sleep Study Sub-Group

☐ I freely agree to participate in this sub study of the MELODY trial as described.

☐ I understand the purposes, procedures and risks of the sub-study described in the project.

☐ I have had an opportunity to ask questions, and I am satisfied with the answers I have received.

☐ I understand that I am free to withdraw from sub study only or the main study at any time and withdrawal will not affect my relationship with any of the organisations and/or study team members.

☐ I give consent storage and use of saliva samples collected during the sub-study for future research project.

Name of Participant (please print) ____

Signature ____________________________ Date ____________________________

Declaration by Study Doctor/Senior Researcher†

I have given a verbal explanation of the research project; its procedures and risks and I believe that the participant has understood that explanation.

Name of Study Doctor/
Senior Researcher† (please print) ____

Signature ____________________________ Date ____________________________

† A senior member of the study team must provide the explanation of, and information concerning, the research project.

Note: All parties signing the consent section must date their own signature.

THE UNIVERSITY OF SYDNEY
Brain and Mind Centre

Withdrawal of Participation Form

**Title**	MELODY Trial: A 12-week randomised controlled trial of adjunctive melatonin or digital cognitive behavioural therapy for insomnia, or placebo to improve depressive symptoms in young adults with mood disorders
**Short Title**	The MELODY Trial
**Protocol Number**	X23-0450
**Project Sponsor**	The University of Sydney
**Principal Investigator**	Professor Ian B. Hickie
**Location**	Brain and Mind Centre, University of Sydney<br>The Woolcock Institute of Medical Research

Declaration by Participant

☐ I wish to withdraw from all participation in the above research project and understand that such withdrawal will not affect my relationship with any of the institutions or clinicians involved in this study. If I wish to withdraw my samples, I may do so by contacting the study team.

☐ I hereby wish to WITHDRAW from the above study, and I am happy for the data already collected to be used.

☐ I hereby wish to WITHDRAW from the above study and request that my data be completely deleted from the study.

Name of Participant (please print) ______________________

Signature ______________________ Date ______________________

Declaration by Sleep Study Sub-Group Participants Continuing in Main Study Activities

I wish to withdraw from participation in the above research project's sub-study activities but continue to participate in the main study activities. I understand that such withdrawal will not affect my relationship with any of the institutions or clinicians involved in this study.

Name of Participant (please print) ______________________

Signature ______________________ Date ______________________

If the participant's decision to withdraw is communicated verbally, the Study Doctor/Senior Researcher will need to provide a description of the circumstances below.

Declaration by Study Doctor/Senior Researcher†

I have given a verbal explanation of the implication of withdrawal from research study, and I believe that the participant has understood that explanation.

Name of Study Doctor/
Senior Researcher† (please print) ______________________

Signature ______________________ Date ______________________
