# Supplementary material for: MELODY trial: study protocol for a 12-week randomised controlled trial of adjunctive melatonin, digital cognitive behavioural therapy for insomnia or pill placebo to improve depressive symptoms in young adults with mood disorders
Source: BMJ Open. 2026 Aug 24;16(8):e114817. doi: 10.1136/bmjopen-2025-114817 (PMC13504852; doi:10.1136/bmjopen-2025-114817)
Supplement: Supplementary file 2 [file bmjopen-2025-114817.reviewer_comments.pdf]

PEER REVIEW HISTORY

BMJ Open publishes all reviews undertaken for accepted manuscripts. Reviewers are asked to complete a checklist review form and are provided with free text boxes to elaborate on their assessment. These free text comments are reproduced below.

ARTICLE DETAILS

Title (Provisional)

MELODY trial: Study protocol for a 12-week randomised controlled trial of adjunctive melatonin, digital cognitive behaviour therapy for insomnia, or pill placebo to improve depressive symptoms in young adults with mood disorders

Authors

Crouse, Jacob J; Shin, Mirim; Hockey, Samuel; Jeon, Elie; Bradshaw, Nathan; Nichles, Alissa; Zmicerevska, Natalia; Chong, Min Kyung; You, Hannah; Wray, Naomi R; Scott, Jan; Grunstein , Ronald; Naismith, Sharon L; Merikangas, Kathleen R; Cain, Sean W; Medland, Sarah E; Iorfino, Frank; Uebergang, Thomas; McKenna, Sarah; Carpenter, Joanne Sarah; Tonini , Emiliana; Bhattacharya, Nayonika; Hamilton, Blake; Wallace, Leanne M; Henders, Anjali K; Scott, Elizabeth M; Song, Yun Ju Christine; Lived Experience Working Group, Brain and Mind Centre; Hickie, Ian B

VERSION 1 - REVIEW

**Reviewer**	**1**
**Name**	**Olmstead, Richard**
**Affiliation**	**University of California Los Angeles**
**Date**	**13-Apr-2026**
**COI**	**None**

Well-written protocol manuscript that appears to cover all the SPIRIT elements (the Appendix did not appear to be accessible from the online review portal).

I assume there is a safety protocol if a participant indicates a risk of harm to self or others to the research team. It would be good to note this in the main protocol document.

**Reviewer**	**2**
**Name**	**Sato, Daisuke**
**Affiliation**	**Nagoya City University Graduate School of Medical Sciences and Medical School**
**Date**	**22-May-2026**
**COI**	**None**

---

1. The manuscript states that the primary planned contrast is the combined active intervention group, that is, the melatonin and dCBT-I arms, versus placebo. However, melatonin and dCBT-I differ substantially in their mechanisms of action, mode of delivery, and feasibility of blinding. Therefore, even if this combined contrast

shows a significant effect, it may remain unclear whether the effect is attributable to melatonin, dCBT-I, or both interventions.

Please provide a clearer justification for using this combined active-versus-placebo contrast as the primary analysis.

2. The manuscript describes the trial as “double-blinded.” However, the randomisation section states that participants and assessors are blinded to medication allocation, namely melatonin versus placebo, but not to allocation of medication versus dCBT-I.

Please revise the description of blinding to specify precisely who is blinded to which allocation.

VERSION 1 - AUTHOR RESPONSE

REVIEWER 1:

R1.1: Well-written protocol manuscript that appears to cover all the SPIRIT elements (the Appendix did not appear to be accessible from the online review portal).

R1.1 Response: We confirm that a completed SPIRIT checklist has been included in the Appendix.

R1.2: I assume there is a safety protocol if a participant indicates a risk of harm to self or others to the research team. It would be good to note this in the main protocol document.

R1.2 Response: We confirm that there are standard operating procedures in place, and we have added the following text to the manuscript:

> “In the case that a participant exhibits high levels of stress or emotional distress, or if there are concerns that a participant is an imminent danger to themselves or others, standard operating procedures (SOPs) are in place which outline required steps (e.g., provide support; assure participant of their safety, voluntary participation and right to withdraw; assess mental state; if indicated, enlist a supervisor to conduct a risk assessment; if indicated, contact the treating clinician; document the incident; etc).” (Page 13, Lines 504-509)

REVIEWER 2:

R2.1: The manuscript states that the primary planned contrast is the combined active intervention group, that is, the melatonin and dCBT-I arms, versus placebo. However, melatonin and dCBT-I differ substantially in their mechanisms of action, mode of delivery, and feasibility of blinding. Therefore, even if this combined contrast shows a significant effect, it may remain unclear whether the effect is attributable to melatonin, dCBT-I, or both interventions. Please provide a clearer justification for using this combined active-versus-placebo contrast as the primary analysis.

R2.1 Response: We have edited the text to clarify the rationale for this design choice:

"The primary planned contrast – active interventions (melatonin and dCBT-I arms) vs placebo – reflect the scientific objectives of the trial and statistical and feasibility constraints. First, the primary aim is to determine whether targeting sleep/circadian processes is an effective antidepressant strategy in young adults with mood disorders. Second, existing meta-analyses report comparable effect sizes for melatonin (SMD=0.39) and dCBT-I (SMD=0.43) on depressive symptoms. The expected difference between the two active arms is very small (SMD < 0.10), which would require a sample of >3,000 participants, which is beyond the feasibility of this trial. We acknowledge that the two active interventions differ in their administration, blinding, and expectancies, and we will examine differences in efficacy in secondary planned contrasts (melatonin vs dCBT-I; melatonin vs placebo; and dCBT-I vs placebo."

(Pages 21-22, Lines 659-669)

R2.2: The manuscript describes the trial as "double-blinded." However, the randomisation section states that participants and assessors are blinded to medication allocation, namely melatonin versus placebo, but not to allocation of medication versus dCBT-I. Please revise the description of blinding to specify precisely who is blinded to which allocation.

R2.2 Response: We have amended the description of the trial design in several locations to be clearer:

"MELODY (Melatonin for Depression in Youth) is an investigator-initiated, single-centre, phase 3, randomised controlled trial with two blinded pharmacological arms (melatonin vs placebo) and one open-label digital intervention arm (digital cognitive behavioural therapy for insomnia [dCBT-I])."

(Page 4, Lines 138-140)

"The MELODY trial is an investigator-initiated, single-centre, phase 3, randomised controlled trial with two blinded pharmacological arms (melatonin vs placebo) and one open-label digital intervention arm (dCBT-I)."

(Page 9, Lines 326-329)

VERSION 2 - REVIEW

Reviewer 2

Name Sato, Daisuke

Affiliation Nagoya City University Graduate School of Medical Sciences and Medical School

Date 26-Jul-2026

COI

The manuscript has been improved and is in a nice condition now.
