# Supplementary material for: MELODY trial: study protocol for a 12-week randomised controlled trial of adjunctive melatonin, digital cognitive behavioural therapy for insomnia or pill placebo to improve depressive symptoms in young adults with mood disorders
Source: BMJ Open. 2026 Aug 24;16(8):e114817. doi: 10.1136/bmjopen-2025-114817 (PMC13504852; doi:10.1136/bmjopen-2025-114817)
Supplement: Supplementary file 3 [file bmjopen-16-8-s001.docx]

**APPENDIX**

**Manuscript:** MELODY trial: Study protocol for a 12-week randomised controlled trial of adjunctive melatonin, digital cognitive behaviour therapy for insomnia, or pill placebo to improve depressive symptoms in young adults with mood disorders

**Table of contents**

1. Table S1. World Health Organization Trial Registration Data Set.
2. Methods: Additional Information

**Table S1. World Health Organization Trial Registration Data Set.**

| **Data category** | **Information** |
| --- | --- |
| Primary registry and trial identifying number | ACTRN12624000017527 |
| Date of registration in primary registry | 10/01/2024 |
| Secondary identifying numbers | N/A |
| Source(es) of monetary or material support | Wellcome Trust |
| Primary sponsor | The University of Sydney |
| Secondary sponsor(s) | N/A |
| Contact for queries | Jacob Crouse PhD (jacob.crouse@sydney.edu.au) |
| Title | MELODY trial: Study protocol for a 12-week randomised controlled trial of adjunctive melatonin, digital cognitive behaviour therapy for insomnia, or pill placebo to improve depressive symptoms in young adults with mood disorders |
| Countries of recruitment | Australia |
| Health condition(s) or problem(s) studied | Major Depressive Disorder and Bipolar Disorder type II |
| Intervention(s) | - Melatonin arm: 2mg immediate-release melatonin, to be taken nightly 2 hours before their desired bedtime. Dose is to be increased to 4mg after 4 weeks, subject to medical screening - Placebo arm: placebo to be taken nightly 2 hours before their desired bedtime, dose also to be doubled after 4 weeks, subject to medical screening - dCBT-I arm: receive dCBT-I (Brand: SHUTi), an online platform for improving sleep. The program provides sleep hygiene training, stimulus control, sleep restriction, relaxation training, cognitive therapy, and relapse prevention over 6 Cores for 12 weeks. |
| Key eligibility criteria | - Aged 18-30 (inclusive at the time of providing consent); and - Current diagnosis of Major Depressive Disorder (MDD) or Bipolar Disorder type II (BD-II), confirmed via the Structured Clinical Interview for DSM-5 (SCID-5)^61^ at the time of enrolment; and - A score of ≥11 on the Quick Inventory of Depressive Symptomatology, Adolescent version, Clinician Rating (QIDS-A-CR), indicating a moderate-to-severe Major Depressive Episodes (MDE), assessed within 14 days prior to study entry; and - Evidence of significant sleep or sleep-wake complaints, indicated by any of the following:   - Moderate-to-severe sleep disturbance, indicated by a T-score ≥ 60 on the Patient-Reported Outcome Measurement Information System (PROMIS^TM^) Sleep Disturbance 8-item short form; or   - Sleep-related impairment, indicated by a T-score ≥ 60 on the Patient-Reported Outcome Measurement Information System (PROMIS^TM^) Sleep-Related Impairment 8-item short form; or   - Irregular sleep-wake timing, according to a response of “Never” or “Rarely” to the following modified item from the RU-SATED scale, “Generally speaking, I go to bed and get out of bed at about the same time every day (within 2 hours), disregarding changes due to social occasions”; or   - Delayed sleep phase, indicated by self-reported habitual sleep onset time ≥ 2 hours later than the desired or required bedtime; and - Ability to provide written informed consent, including both adequate intellectual capacity and fluency in English |
| Study type | Randomised controlled trial |
| Date or first enrolment | August 2025 |
| Target sample size | 660 |
| Recruitment status | Recruiting |
| Primary outcome(s) | Change in depressive symptoms from baseline to 12 weeks, as assessed by the Quick Inventory of Depressive Symptomatology (QIDS; Adolescent version) – Clinician Rated |
| Key secondary outcome(s) | - Partial remission of major depressive episode (MDE) at 12 weeks according to SCID-5 - Change in depressive symptoms from baseline to 12 weeks, assessed by the PHQ-9 and QIDS-A-SR - Change in anxiety symptoms from baseline to 12 weeks, assessed by the GAD-7 and the OASIS - Change in anxiety/depressive symptoms from baseline to 12 weeks, assessed by the RCADS (18-year-olds only) - Change in insomnia symptoms from baseline to 12 weeks, assessed by the ISI - Change in general sleep-related impairment from baseline to 12 weeks, assessed by the PROMIS Sleep-Related Impairment short form 8a - Change in general sleep disturbance from baseline to 12 weeks, assessed by the PROMIS Sleep Disturbance short form - Change in chronotype from baseline to 12 weeks, assessed by the MEQ - Change in quality of life from baseline to 12 weeks, assessed by the ReQoL - Change in social and occupational functioning from baseline to 12 weeks, assessed by the SOFAS - Change in disability from baseline to 12 weeks, assessed by the WHODAS 2.0 - Change in suicidal thoughts and behaviours from baseline to 12 weeks, assessed by the SIDAS - Change in hypo/manic symptoms from baseline to 12 weeks, as assessed by the ASRM - Change in alcohol, cannabis, and tobacco use from baseline to 12 weeks, assessed by the WHO-ASSIST - Change in sleep-waking timing from baseline to 12 weeks, assessed by the Consensus Sleep Diary - Change in sleep-wake cycles from baseline to 12 weeks, assessed by actigraphy - Change in dim-light melatonin onset (DLMO) time from baseline to 12 weeks, as measured by salivary melatonin sampling (20% subset of cases) - Change in sleep physiology (exploratory) from baseline to 12 weeks, assessed by Sleep Profiler^TM^ EEG Sleep Monitor (20% subset of cases) |

**Methods: Additional Information**

**Ancillary and post-trial care**

If a participant suffers any injuries or complications as a result of the study, the Study Doctor will assist them in arranging appropriate medical treatment. They will also have a right to take legal action to obtain compensation for any injuries or complications resulting from the study. Compensation may be available if their injury or complication is sufficiently serious and is caused by unsafe drugs or equipment, or by the negligence of one of the parties involved in the study.

**Withdrawal/discontinuation**

**Voluntary Withdrawal by Participant**

- Participants may withdraw from the study at any time without penalty or loss of entitled compensation.
- If a participant expresses a wish to withdraw:
  - A member of the study team will discuss the reason for withdrawal and answer any questions.
  - The participant will be asked (but not required) to complete a withdrawal form.
  - The participant will be informed that they can choose to withdraw from:
    - Further study participation (including follow-ups and assessments).
    - Data collection and use of previously collected data.
- Participants will be asked if they consent to continued use of already collected data for research purposes.

**Investigator-Initiated Withdrawal**

- A participant may be withdrawn from the study at the discretion of the investigator if:
  - They develop a medical condition that makes continued participation unsafe.
  - They experience a serious adverse event (SAE) related to the study intervention.
  - They fail to comply with the study protocol (e.g., non-adherence to medication or dCBT-i).
  - There are safety concerns identified by the study doctor.
- The study doctor will assess the participant’s health and safety before withdrawal.
- The reason for withdrawal will be documented in the trial database.

**Administrative Withdrawal**

- Participants may be withdrawn for administrative reasons such as:
  - Loss of contact.
  - Relocation.
  - Study termination.
- Administrative withdrawals will be documented in the trial database.

**Emergency unblinding**

Criteria for Emergency Unblinding:

- A serious adverse event (SAE) or medical emergency occurs where knowledge of the assigned intervention is necessary for appropriate medical treatment.
- The study doctor determines that knowledge of the intervention is required to guide clinical management.

Process for Emergency Unblinding:

- The study doctor must complete an *Unblinding Request Form* detailing:
  - Participant ID
  - Reason for unblinding request
  - Clinical justification for unblinding
  - Date and time of request
- The study doctor will contact the Clinical Research Manager to request unblinding.
- The Clinical Research Manager will contact unblinded study staff to access the unblinding code on REDCap Randomisation.
- The unblinded study staff will inform the study doctor of the intervention allocation.
- The PI will be notified of the unblinding within 24 hours.
- The participant’s medical management will be adjusted accordingly.

Criteria for Non-Emergency Unblinding:

- The trial reaches its planned conclusion.
- The participant voluntarily withdraws from the study and requests to know their assigned intervention.
- The Data and Safety Monitoring Board (DSMB) or regulatory authority requests unblinding for safety review purposes.

Process for Non-Emergency Unblinding:

- The PI will approve any non-emergency unblinding request.
- The Clinical Research Manager will access the unblinding code through the designated secure platform.
- The participant will be informed of their assigned intervention by the study doctor if appropriate.
- The study team will continue to collect follow-up data from the participant unless they have withdrawn consent.

**Reimbursement and incentives**

Participants will receive $150 in financial compensation, in the form of vouchers, paid out as follows:

- $50 for completion of all baseline activities, including return of actigraphy watch
- $50 for completion of intervention & end of treatment visit, including return of actigraphy watch
- $50 for completion of follow-up activities, including return of actigraphy watch

Participants in the laboratory sub-study will receive an additional $200 for completion of all sleep study activities.
